# Effect of point-of-purchase calorie labeling on restaurant and cafeteria food choices: A review of the literature

**DOI:** 10.1186/1479-5868-5-51

**Published:** 2008-10-26

**Authors:** Lisa J Harnack, Simone A French

**Affiliations:** 1 Division of Epidemiology and Community Health, School of Public Health, University of Minnesota, 1300 South 2nd St Suite 300, Minneapolis, MN 55454, USA

## Abstract

**Background:**

Eating away from home has increased in prevalence among US adults and now comprises about 50% of food expenditures. Calorie labeling on chain restaurant menus is one specific policy that has been proposed to help consumers make better food choices at restaurants. The present review evaluates the available empirical literature on the effects of calorie information on food choices in restaurant and cafeteria settings.

**Methods:**

Computer-assisted searches were conducted using the PUBMED database and the Google Scholar world wide web search engine to identify studies published in peer-review journals that evaluated calorie labeling of cafeteria or restaurant menu items. Studies that evaluated labeling only some menu items (e.g. low calorie foods only) were excluded from the review since the influence of selective labeling may be different from that which may be expected from comprehensive labeling.

**Results:**

Six studies were identified that met the selection criteria for this review. Results from five of these studies provide some evidence consistent with the hypothesis that calorie information may influence food choices in a cafeteria or restaurant setting. However, results from most of these studies suggest the effect may be weak or inconsistent. One study found no evidence of an effect of calorie labeling on food choices. Each of the studies had at least one major methodological shortcoming, pointing toward the need for better designed studies to more rigorously evaluate the influence of point-of-purchase calorie labeling on food choices.

**Conclusion:**

More research is needed that meets minimum standards of methodological quality. Studies need to include behavioral outcomes such as food purchase and eating behaviors. Also, studies need to be implemented in realistic settings such as restaurants and cafeterias.

## Introduction

Eating out has become increasingly common in the US [1], with Americans now spending almost half of their food dollars on foods away from home [2]. Food eaten away from home at fast food and other restaurants has garnered particular scientific interest recently because it is associated with higher energy, fat and saturated fat intake; lower intake of fiber and calcium; greater consumption of hamburgers, French fries and soft drinks, and lower fruit and vegetable intake [1,3-9]. Several prospective studies have shown that frequent eating away from home at restaurants, especially fast food restaurants, is associated with excess weight gain over time, compared to infrequent restaurant use [3,4,10-13]. In the largest and longest study conducted to date, frequent fast food restaurant eating was associated with significantly greater weight gain among 3031 adults over a 15 year follow-up period [10,11].

**Figure 1:**
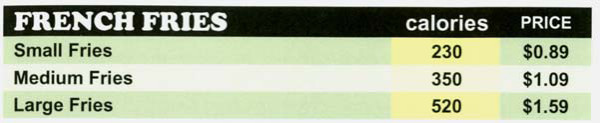
An example of a portion of a restaurant menu with calorie information provided for menu items.

Calorie labeling on chain restaurant menu boards is one public health policy that has been proposed to help consumers make better food choices at restaurants [14]. To elaborate, it has been argued that consumers need to be informed about the calories in the menu items at restaurants because without this information they may have little awareness or the number of calories in the foods they are purchasing. It is thought that such information is most effective at the point of choice, and therefore should be displayed on menus or menu boards next to the food items. An example of this type of calorie labeling format is provided in Figure 1.

What is known about the effect of calorie labeling on restaurant meal choices? The intent of this paper is to review published research on the effect of point-of-purchase calorie labeling on cafeteria and restaurant menu food choices. Following the literature review the findings are synthesized with reference to the broader conceptual model of the restaurant food choice process. Recommendations for the design of new research are made in light of the conceptual model and the available studies. It is hoped that the review of the scientific findings may inform policy development and implementation in the area of food labeling in restaurant settings.

## Methods

In accord with guidelines for conducting and reporting systematic reviews [15] a structured approach was utilized to identify, review, and draw conclusions from studies that have examined the effect of point-of-purchase calorie labeling on food choices. With regard to identifying relevant studies, computer-assisted searches were conducted using the PUBMED database and the Google Scholar World Wide Web search engine. These searches were conducted in February and March of 2008 using various combinations of the following key words: nutrition labeling, calorie labeling, nutrition education, point-of-purchase, restaurant, cafeteria, and fast food. Articles were also identified from references from published research and reviews.

Studies that evaluated labeling only some menu items (e.g., labeling low calorie foods only) were excluded from the review since the influence of selective labeling may be different from that which may be expected from comprehensive labeling. In addition, studies evaluating calorie labeling in settings other than restaurants or cafeterias were excluded from the review because consumers may consider a different set of factors when purchasing food from grocery stores versus purchasing a meal from a restaurant or cafeteria. The review was also restricted to studies written in English and reported in peer reviewed publications.

A complete copy of each article meeting study inclusion criteria was obtained and reviewed by each author (LJH, SAF). Key information about each study (e.g. study aims, study design, sample size, etc.) were recorded on a spreadsheet to facilitate comparing and summarizing study results.

## Results

Six studies were identified that met the selection criteria for this review [16-21]. To summarize, results from five of these studies provided some evidence consistent with the hypothesis that calorie information may influence food choices in a cafeteria or restaurant setting [16-20]. However, results from most of these studies suggest a weak or inconsistent effect. One study found no evidence of an effect of calorie labeling on food choices [21]. Each of the studies had at least one major methodological shortcoming, pointing toward the need for better designed studies to more rigorously evaluate the influence of point-of-purchase calorie labeling on food choices.

### Studies that Reported Significant Effects

Cinciripini et al. evaluated the influence of calorie labeling in a University cafeteria frequented by undergraduate students using an ABA experimental design [18]. During an eight week pre-labeling period trained observers recorded the food selections of cafeteria patrons. Calorie labeling was then implemented for eight weeks during which time food selections were recorded by trained observers. The labeling involved listing the calorie information for menu items on two large signs placed on tripods at each entrance of the cafeteria. Leaflets were distributed to cafeteria patrons during the first ten days of the labeling period to draw attention to the signs and explain their use. After labeling was discontinued (post-labeling period), the food selections of cafeteria patrons were observed for an eight week period. Selections of foods in seven categories were analyzed stratified by sex and bodyweight status. Results indicated that selection of carbohydrate rich foods (e.g. breads and starchy vegetables) was statistically significantly lower during the labeling period compared to baseline for all sex and bodyweight groups. No other food group was significantly different during the labeling period across all sex and bodyweight groups. However, red meat selection was significantly lower during calorie labeling in all groups except overweight females. Selection of regular-fat dairy products was significantly lower during the labeling period among normal weight males and normal weight females only. Selection of high fat foods/desserts/sauces and vegetable/soup/fruit/low-fat dairy groups was significantly lower only among overweight females. Selection of salads was higher during the calorie labeling versus baseline period among lean men. No significant differences in selection were found for the chicken/fish/turkey food category. The effect of labeling on the energy content of meals selected was not examined.

Balfour et al. evaluated the effect of computerized nutrition information on food choices in cafeterias in two worksite settings, one located in a hospital and the other within the corporate office of an oil company [17]. Customers entering each cafeteria were invited to choose their meal using a computer system that displayed the calories, saturated fat, fiber, and added sugar content of meals selected. After the nutrition feedback about the initial meal choice was received a second meal choice could be made if desired. In this study only approximately 45% of those entering the cafeterias agreed to use the system. Among those using the system 17% at the oil company and 15% at the hospital changed their meal choice in response to the nutrition information. Among this subset the average energy composition of the second meal selection was significantly lower than the first meal choice. For example, among those who made a second meal choice at the cafeteria located with a corporate office building the average energy content of the meal selected initially was 711 kcal compared to 606 kcal for the second meal choice.

Using an AB experimental design Milich et al. evaluated the effect of calorie labeling on food choices among 450 women eating at a hospital cafeteria [19]. Following a two week baseline period during which food choices of women eating at the cafeteria were recorded by trained observers food prices in the cafeteria were unexpectedly increased. To cope with this unexpected change food choices were observed for one week following this price change, and then calorie labeling was implemented with food choices observed for an additional one week period. The calorie labels were placed as close as possible to food items sold in the cafeteria. The labels consisted of a 5 cm × 5 cm card on which calories were printed in red ink. The average calorie composition of meals selected during the calorie labeling period (459 kcal/meal) was significantly lower than that of the baseline (507 kcal/meal) and price increase (525 kcal/meal) periods (p < 0.02). Results were found to be similar across bodyweight category.

In contrast to the other studies just described which focused on adults, Yamamoto et al. evaluated the effect of calorie labeling on restaurant food choices in a sample of adolescents recruited from a middle school and high school [16]. Each participant (n = 106) was asked to order a dinner meal of their choice from three different restaurant menus (McDonald's, Panda Express, and Denny's). These mock menu orders were recorded by study staff and then the participants were shown a version of each menu that included calorie and fat content information for all menu items. After viewing the calorie and fat labeled menus participants were asked if they would like to change their meal order. If they responded affirmatively their new order was recorded. A modest fraction of participants (31of 106) changed one or more of their meal orders when shown the menus with calorie and fat information. In total, 54 meal orders were modified, with 43 modified in a way that resulted in a lower calorie meal and 11 modified in a manner that resulted in a higher calorie meal relative to the initial meal choice.

The final study that reported results in support of calorie labeling was a mail survey conducted to examine whether the provision of nutrition information would influence consumers' attitudes and purchase intentions for restaurant menu items [20]. In this study 482 adults in a south-central state were mailed a packet that included 1 of 6 randomly assigned menus and a survey that included a series of questions related to the menus. The six menus sent varied with respect to the type of nutrition information included (calories, fat, saturated fat, *trans *fat, and sodium; calories only; no nutrition information) and whether daily value information was included (included or excluded). All of the menus contained the following four menu items: hamburger platter, chef's salad, chicken breast dinner, and turkey sandwich. It was hypothesized that providing calorie information that is inconsistent with people's expectation would decrease their purchase intentions for the high calorie foods. For example, purchase intentions should be lower for the burger and for the Chef salad because the calories were higher than people expected. Completed surveys were returned by 241 adults resulting in a response rate of 50%. Results indicated that for two of the four menu items, reported purchase intentions were significantly different between those who received the calories only menu relative to those who received a menu without nutrition information. Hamburger platter purchase intentions were lower and turkey sandwich purchase intentions were higher among those who received the calorie-only menu compared to those who received a menu without nutrition information. Purchase intentions for the chicken meal and chef salad were comparable between these two groups. These results are thus mixed with regard to the expectation. It was initially hypothesized that orders for chef salad would decline when participants were given information that showed the high calorie content of the salad. Limitations of the study were that only four food items were included and there was no initial a priori measure of people's "expected" calories for each of the items. Consequently, it is not known whether any inconsistency was actually created among participants by presenting the calorie information.

### Study that Reported No Effect of Calorie Labeling on Food Choices

Mayer et al. evaluated the influence of calorie labeling on food choices in the cafeteria of a Fortune 500 company office building using an ABA experimental design [21]. Food items selected by cafeteria patrons were recorded by trained observers during a four week baseline period. Following this period the calorie content of all food items were listed on index cards placed near foods. Along with this labeling a nutrition awareness game was implemented and raffles were held one day per week to encourage selection of three lower calorie menu items. The labeling and promotional activities occurred over a four week period during which trained observers recorded the meal choices of patrons. The labeling and promotions activities were then discontinued, and meal choices of patrons were recorded by the observers for four weeks post-intervention. The mean number of calories per tray during each experimental phase was similar (462, 454, and 464 calories during each period respectively; p > 0.38). Sample sizes and participation rates were not included in the published report.

## Discussion

Results from five of the six studies included in this review provide some support for the supposition that calorie information may have a positive influence (i.e., fewer calories purchased or selected) on food choices in a cafeteria or restaurant setting. It is important to note though that the magnitude of the effects seen tended to be small. Also, results were inconsistent in some studies. For example, Burton et al. found purchase intentions to be affected by calorie labeling for just two of the four foods included on the study menu (21) and Yamomato et al. found that only about 20% of intended food orders were modified following provision of calorie information for restaurant menu items (19).

Conceptual models of food choice behaviors often consider a broader time frame of food decision-making and include broader contextual effects such as family relationships, age and life course [22-24]. Sobal and colleagues found that people often explain current food choices in terms of both past experiences and current situations [22]. For example, a person with a lifelong history of eating vegetables may make different food choices at a restaurant than a person who only recently began eating vegetables. Personal influences, such as physiological, psychological, and emotional factors; resources such as money, time, transportation and skills; and social factors such as relationships, families, and roles; and contexts such as households and neighborhoods, are some of the levels at which food choices may be influenced. These influences operate through individual level personal food systems, which include the personal values people place on factors such as taste, convenience, cost, health and managing personal relationships. When viewed in the context of broader conceptual models of the food choice decision-making process, the apparent limited effect of calorie labeling on food choices may reflect the variety of factors beyond nutrition information that influence food purchase decisions. Results from recent studies suggest that factors such as taste, price, convenience and social relationships tend to be rated as more important considerations than nutrition when making restaurant meal choices [25,26]. For example, among a convenience sample of adults who eat at fast food restaurants regularly 57.9% rated nutrition as very important or somewhat important when selecting foods from a fast food restaurant. In contrast, 96.1%, 89.6% and 87.2% rated taste, convenience, and price as important or very important, respectively [25].

The effect of point-of-purchase calorie labeling on food choices could possibly be strengthened if the weight given to this information and its expected outcome is increased. For example, the value of considering calories when making food choices at restaurants could be strengthened through promotional messages combined with the calorie labels. Several studies provide support for this supposition [27-29]. For example, in a study evaluating the effect of calorie labeling on vending machines sales Bergen et al. found labeling to have an effect on sales only when accompanied by a promotional poster [28]. Likewise French et al. found low-fat labeling in vending machines to influence sales only when the labeling was provided in tandem with an educational poster [27]. These results suggest that modest promotional efforts may prompt consumers to give nutrition information greater consideration in the food selection process.

It is important to note that the studies included in this review have a number of significant methodological shortcomings. First and foremost, four of the six studies evaluated calorie labeling in worksite [17,19,21] or university [18] cafeteria settings. Nutrition information provided in a restaurant setting may be utilized differently than information provided in a cafeteria setting because individuals may consider a different set of factors when they select foods from a restaurant versus an employee cafeteria. For example, eating at a restaurant may be viewed as an occasion to treat oneself or splurge (e.g., You Deserve a Break Today™) whereas moderation may be a greater consideration when eating in a cafeteria. Two of the studies evaluated calorie labeling on restaurant menus [16,20]. However, both measured intended rather than actual food choices. Consequently, social desirably bias in reporting is a significant concern in these studies. "Simulation" studies of intended or hypothetical food choices also fail to incorporate the social nature of food choices and economic factors that might influence food choices. Also, food choices might occur at the restaurant level, not at the food item level within the restaurant. For example, a person whose food choice is barbequed ribs would probably not choose to go to McDonalds for a meal, so the food choice itself may be made prior to arriving at the restaurant and forms the basis for the choice of restaurant. Other major weaknesses of the studies reviewed include use of quasi-experimental designs [17-19,21] where factors other than the experimental conditions being tested may have differed across test periods due to lack of randomization.

## Conclusion

Better designed studies to more rigorously evaluate the influence of point-of-purchase calorie labeling on restaurant food choices are needed. Ideally experimental studies measuring actual food choices in restaurant settings would be conducted, thus maximizing both internal and external validity of results. However, it may be difficult to find restaurants willing to participate in these types of studies due to concern that the type of menu manipulation to be evaluated may have an adverse effect on revenue. Thus, consideration should be given to designing quasi-experimental studies in municipalities or regions where mandatory calorie labeling regulations have been implemented. For example, the implementation of a mandatory restaurant calorie labeling rule in New York City in 2008 [30] presents opportunities for evaluating calorie labeling in a naturalistic setting.

Despite the methodological limitations of the studies included in this review, results across studies uniformly indicate that calorie labeling may have a beneficial effect on food choices made away from home. However, the effect is likely limited in magnitude. This limited effect may reflect the low level of importance many consumers place on nutrition when eating out. It may also reflect the multi-level nature of food choices, with influences occurring at the individual level prior to the restaurant, and other strong environmental influences at the restaurant, such as food choices, prices and other promotional activities at the point-of-purchase, and the influence of other people at the point of choice. Multiple levels of influence may need to be targeted in tandem, including consumer attitudes about calories when eating out, in order for calorie labeling to have a more substantial influence on restaurant food choices.

## Competing interests

The authors declare that they have no competing interests.

## Authors' contributions

LH conducted the literature search. LH and SF reviewed each of the articles. Both authors also drafted the manuscript and approved the final version.
